# Longitudinal Assessment of Amyloid-β Deposition by [18F]-Flutemetamol PET Imaging Compared With [11C]-PIB Across the Spectrum of Alzheimer’s Disease

**DOI:** 10.3389/fnagi.2019.00251

**Published:** 2019-09-11

**Authors:** Shizuo Hatashita, Daichi Wakebe, Yuki Kikuchi, Atsushi Ichijo

**Affiliations:** ^1^Department of Neurology, Shonan-Atsugi Hospital, Atsugi, Japan; ^2^Department of Radiology, Shonan-Atsugi Hospital, Atsugi, Japan; ^3^Department of Radiopharmacology, Shonan-Atsugi Hospital, Atsugi, Japan

**Keywords:** Alzheimer’s disease, amyloid imaging, PET, amyloid beta, flutemetamol

## Abstract

This study evaluates the longitudinal changes in the amyloid-β (Aβ) deposition with [18F]-flutemetamol (FMM) PET imaging across the spectrum of Alzheimer’s disease (AD), compared with [11C]-Pittsburgh Compound-B (PIB) PET. Eleven AD, 17 mild cognitive impairment (MCI) and 13 cognitively normal (CN) subjects underwent neuropsychological assessment and amyloid PET imaging using [18F]-FMM and [11C]-PIB during a follow-up period. Regions of interest were defined on co-registered MRI, and the FMM and PIB standardized uptake value ratio (SUVR) was used in the same cortical regions. The annual rate of change in FMM and PIB SUVRs was calculated. Cortical FMM SUVR in amyloid-positive subjects increased over a follow-up of 3.1 ± 0.5 years. An individual FMM SUVR was significantly correlated with PIB SUVR at baseline and at follow-up in the same AD, MCI, and CN subjects. The annual rate of increase in FMM SUVR was significantly greater in typical amyloid-positive (0.033 ± 0.023, *n* = 7), focal positive MCI (0.076 ± 0.034, *n* = 4) and positive CN (0.039 ± 0.027, *n* = 4) while that in AD (0.020 ± 0.018, *n* = 11) was smaller. Among amyloid-positive patients, the baseline FMM SUVR was inversely related with the increased rate in FMM SUVR (r=−0.44, *n* = 26, *p* < 0.05). An individual annual rate in change of cortical FMM SUVR was significantly correlated with that in cortical PIB SUVR. Our results suggest that the [18F]-FMM PET imaging can clarify the longitudinal assessment of Aβ deposition across the AD spectrum, similarly to [11C]-PIB PET. The Increase in Aβ deposition is faster in the predementia stage but not at a constant rate across the clinical stages of the AD spectrum.

## Introduction

The National Institute on Aging Alzheimer’s Association (NIA-AA) workgroup has proposed diagnostic criteria for the spectrum of Alzheimer’s disease (AD) supported by biomarkers of the underlying pathophysiological process (Jack et al., 2011). This disease framework for AD with biomarkers is an important advance in clinical and pathophysiological progression. Among the biomarkers, amyloid PET imaging is the biomarker of the amyloid-β (Aβ) plaques which represents the earliest evidence of AD neuropathological change currently detectable in living persons and determines whether individuals are on the AD spectrum. Amyloid PET imaging is a key approach for the AD spectrum in general clinical practice.

Amyloid PET imaging with [11C]-labeled Pittsburgh Compound-B (PIB), which has a high affinity for fibrillar Aβ, detects Aβ deposition in the brain and is a distinctive and reliable Aβ biomarker. The [11C]-PIB PET imaging has demonstrated the usefulness of assessing the Aβ plaque status of subjects and has been well established (Klunk et al., 2004; Hatashita and Yamasaki, 2010). [11C]-PIB PET imaging has been extensively used in clinical research, trial and practice for AD. However, [11C]-PIB PET tracer can only be used in large PET centers with their own on-site cyclotron and radiopharmacy facilities due to the 20 min half-life of [11C]. Following the success of [11C]-PIB, several fluorine-18 [18F]-labeled Aβ-selective radiopharmaceuticals have been developed for clinical purposes. They are more suitable radioisotopes for more routine clinical usefulness because the 110 min half-life of [18F] allows distribution from a production site to multiple PET centers. [18F]-labeled amyloid PET imaging has been recently approved and has replaced [11C]-PIB PET imaging (Morris et al., 2016). In particular, [18F]-flutemetamol (FMM) is a fluorinated derivative of [11C]-PIB and is structurally identical to [11C]-PIB. [18F]-FMM PET imaging has been demonstrated to reliably detect Aβ deposition in the brain with imaging-to autopsy comparison, and distinguish AD patients from healthy controls (HC) subjects with both visual reads and quantification in the cortical regions, similar to [11C]-PIB PET imaging (Ikonomovic et al., 2008; Hatashita et al., 2014).

The longitudinal assessment of the increasing amyloid accumulation during the AD process is an important aspect of the clinical progression of the disease across the AD spectrum. Some longitudinal studies with [11C]-PIB PET imaging have shown that Aβ deposition increases continuously from levels in HC to those in AD dementia, and Aβ deposition slows in the later stages of AD (Jack et al., 2013; Villemagne et al., 2013). In contrast, using [18F]-FMM PET imaging, the longitudinal change of Aβ deposition has not yet been successfully clarified across the AD spectrum. It is still unknown whether the clinical progression of the disease across the spectrum of AD is associated with the amount in Aβ deposition and/or the rate of increase in Aβ deposition.

The aim was to evaluate the longitudinal change in the Aβ deposition across the spectrum of AD using amyloid PET imaging with [18F]-FMM and comparing this with [11C]-PIB. We sought to clarify the relationship between the amount of Aβ deposition, the annual rate of change in Aβ deposition, and the clinical progression of the disease.

## Materials and Methods

### Subjects

Forty one subjects aged 60–90 years were recruited from our memory clinic and a community advertisement, and then included in a longitudinal study. Some subjects had participated in our earlier baseline study (Hatashita et al., 2014). All subjects underwent neurological and neuropsychological assessment, and amyloid PET imaging using [18F]-FMM and [11C]-PIB at baseline and one or more during the 3.1 ± 0.5 years of follow-up (range: 2.5–4.5 years). Global cognitive status was assessed with the Mini-Mental-State Examination (MMSE; Folstein et al., 1975) and the severity of dementia was rated on the Clinical Dementia Rating (CDR) scale and CDR sum of boxes (CDR SB; Morris, 1998). Memory measurement of immediate and delayed recall of a paragraph from the Wechsler Memory Scale-Revised (WMS-R) Logical Memory II was performed as a simple episodic memory test (Wechsler, 1987). The apolipoprotein E (APOE) genotype was determined at baseline.

Of these participants, 11 patients with AD dementia met the core clinical criteria of the NIA-AA for probable AD (McKham et al., 2011). The MMSE score was less than or equal to 23 and a CDR score was greater than 0.5. Seventeen patients with mild cognitive impairment (MCI) met the core clinical criteria for MCI by the NIA-AA (Albert et al., 2011), including an MMSE score greater than or equal to 24 and a global CDR score of at least 0.5 in the memory domain. Thirteen cognitively normal (CN) subjects had normal cognitive function with a MMSE score of 28 or greater and a global CDR score of 0. Participants were excluded if they had other systemic or brain diseases, including degenerative, vascular, depressive, traumatic, medical comorbidities, mixed disease, or traumatic brain injury.

The study was approved by the Ethics Committee of the Mirai Iryo Research Center Incorporation (Tokyo, Japan). All subjects or their caregivers provided written informed consent for participation.

### PET Imaging

The [18F]-FMM and [11C]-PIB were produced in our PET center with good manufacturing practice guidelines (PIC/S GMP Guide Annex 3) according to standard procedures, as previously described (Hatashita et al., 2014).

All subjects underwent a [11C]-PIB PET scan on the same day as the cognitive testing, and a [18F]-FMM PET scan on the next day. PET imaging was conducted using a Siemens ECAT ACCEL scanner with an axial field of view of 155 mm, providing 63 contiguous 2.4 mm slices with a 5.6mm transaxial and a 5.4 mm axial resolution. Images were reconstructed with an iterative reconstruction algorithm, using a Gaussian filter of 3.5 mm full-width at half-maximum. The subject’s head was immobilized to minimize motion during the scan. In each case, 10 min of transmission data were acquired for attenuation and scatter correction before the emission scan.

The [11C]-PIB was injected intravenously as a bolus with a mean dose of 550 ± 10% MBq. Dynamic PET scanning in the three-dimensional mode was performed for 60 min using a predetermined protocol. A single dose of [18F]-FMM of 190 ± 10% MBq was injected as a bolus. The image acquisition window of the [18F]-FMM extended from 85 to 115 min. All subjects underwent T1-weighed MRI (1.5 Tesla) for screening and subsequent co-registration with the PET images.

### Image Analysis

A region of interest (ROI) analysis was performed on individual PET images. MRI-based correction of the PET data for partial volume effects was carried out using the PMOD software (PMOD Technologies Limited, Adliswil, Switzerland). The ROIs were manually drawn on the co-registered MRI in each subject and included the following 20 bilateral cortical regions: lateral temporal cortex (LTC), medial temporal cortex (MTC), frontal cortex (FC), occipital cortex (OC), parietal cortex (PC), sensory motor cortex (MC), anterior cingulate gyrus (ACG), posterior cingulate gyrus (PCG), precuneus cortex (Pre) and cerebellar cortex. The cerebellar gray matter was used as a reference region. The ROIs of the follow-up PET images were co-registered with the initial PET images, and the same ROIs were applied to both the baseline and follow-up images.

The retention of [18F]-FMM was calculated as the regional-to-cerebellum standardized uptake value ratios (SUVR). The regional FMM SUVR in each cortical region and cortical FMM SUVR for the mean of the regional SUVR over the nine cortical regions, including LTC, MTC, FC, OC, PC, MC, ACG, PCG, and Pre, were defined. The retention of [11C]-PIB was calculated as SUVR for 35–60 min. Regional and cortical PIB SUVR values were defined in the same regions as the FMM SUVR.

The cut-off values of FMM SUVR and PIB SUVR for amyloid positivity were based on the bimodal distribution in 56 CN controls and 32 AD patients. The cut-off value of FMM SUVR was 1.36 in cortical region while that of PIB SUVR was 1.39, as previously described (Hatashita et al., 2014). A typical amyloid-positive scan had more than cut-off values of cortical FMM and PIB SUVRs, and of regional FMM and PIB SUVRs in at least four cortical regions of LTC, FC, PC and Pre. The focal amyloid-positive scan had more than the cut-off value of regional FMM and PIB SUVRs in at least one or two regional cortical regions. An amyloid-negative scan had less than the cut-off value of regional FMM and PIB SUVRs in all of the cortical regions.

### Data Management

The subjects underwent clinical assessments and [18F]-FMM PET and [11C]-PIB PET imaging approximately 12 months apart during the follow-up period. Annual changes in the [18F]-FMM and [11C]-PIB SUVRs of each cortical region were calculated for each subject at the last follow-up visit using the following equation: annual change = [(SUVR at last follow-up − SUVR at baseline)/follow-up period (year)].

### Statistical Analysis

Data were analyzed with Statcel 3 software (OMS Inc., Tokyo, Japan). Paired *t*-tests were used to study changes between baseline and follow-up data. Clinical group differences were evaluated with two-sample Student’s *t*-tests. Multiple comparisons of the difference in cortical regions were performed using Bonferroni *post hoc* test. Pearson’s correlation analyses were conducted among the FMM SUVR, PIB SUVR, and clinical features. Categorical variables were examined with Fisher’s exact test. Results were considered significant at *p* < 0.05. Data were presented as means ± standard deviations (SD).

## Results

### Amyloid Positivity

All the 11 subjects with AD dementia had typical amyloid-positive scans. Eleven of the 17 MCI patients were amyloid-positive while six patients were amyloid-negative. Seven of the 11 amyloid-positive MCI patients had typical positive scans and four patients had focal positive scans. Two of four focal positive MCI patients were amyloid-positive in both precuneus and parietal or frontal cortical regions while two were only in precuneus. Four of the 13 CN subjects had typical amyloid-positive scans, and nine subjects were amyloid-negative.

### Clinical Data and Cognitive Function

The demographic characteristics of the AD, MCI and CN subjects at baseline and follow-up are shown in Table 1. There was no difference in a mean age, education level, and sex among these groups. An APOEε4 allele was present in 6 (54%) of 11 AD patients, 6 (54%) of 11 amyloid-positive MCI patients and two (50%) of four amyloid-positive CN subjects. The proportion of APOEε4 carriers in these amyloid-positive subjects was larger than that in the amyloid-negative MCI or CN subjects.

**Table 1:** Demographic characteristics of Alzheimer’s disease (AD), mild cognitive impairment (MCI) and cognitively normal (CN) subjects at baseline and follow-up.

	AD	MCI typ	MCI foc	MCI−	CN+	CN−
*n*	11	7	4	6	4	9
Female	8 (73%)	4 (57%)	3 (75%)	2 (33%)	3 (75%)	6 (66%)
Age (years)	71.1 ± 8.3	76.2 ± 3.1	67.4 ± 4.9	75.0 ± 6.0	69.2 ± 2.3	66.5 ± 4.4
Education (years)	11.2 ± 2.0	11.7 ± 2.1	11.2 ± 1.3	12.3 ± 2.8	12.5 ± 0.8	13.7 ± 1.7
APOEε4 car	6 (54%)	3 (43%)	3 (75%)	1 (16%)	2 (50%)	2 (22%)
at baseline
MMSE	21.0 ± 2.1	26.1 ± 1.4	27.5 ± 1.5	27.5 ± 1.6	29.2 ± 0.8	29.6 ± 0.6
CDR	0.8 ± 0.2	0.5	0.5	0.5	0	0
CDR SB	2.8 ± 1.1	0.7 ± 0.2	0.6 ± 0.2	0.5 ± 0.1	0	0
Imm rec	2.4 ± 2.1	5.5 ± 3.8	7.2 ± 2.0	7.8 ± 4.1	13.0 ± 1.8	15.1 ± 2.8
Del rec	0.1 ± 0.2	1.4 ± 2.6	3.5 ± 2.5	5.3 ± 3.9	11.5 ± 2.1	13.4 ± 2.8
at last follow-up
MMSE	16.1 ± 3.2*	22.0 ± 0.8*	26.5 ± 2.0	26.1 ± 1.7	29.5 ± 0.8	29.6 ± 0.6
CDR	1.3 ± 0.4*	0.7 ± 0.2	0.5	0.5	0	0
CDR SB	5.5 ± 2.5*	2.1 ± 0.9*	0.6 ± 0.2	0.7 ± 0.3	0	0
Imm rec	0.8 ± 0.9	2.2 ± 1.7	6.5 ± 4.5	6.6 ± 4.6	12.0 ± 1.0	15.2 ± 3.5
Del rec	0.1 ± 0.3	0	4.7 ± 3.5	5.0 ± 4.4	9.7 ± 2.0	13.6 ± 4.0
Foll-up (years)	3.3 ± 0.3	3.0 ± 0.2	3.0 ± 0.1	3.0 ± 0.5	3.0 ± 0.3	3.5 ± 0.4

typ, typical amyloid-positive; foc, focal amyloid-positive; +, amyloid-positive; −, amyloid-negative; n, number of patients; APOEε4 car, apolipoprotein Eε4 carriers; MMSE, Mini-Mental State Examination; CDR, Clinical Dementia Rating; Imm rec, WMS-R Immediate recall score; Del rec, WMS-R Delayed recall score; Foll-up, follow-up; data are presented as means ± SD. *Statistically significant difference from baseline by paired *t*-test (*p* < 0.05).

The 11 AD patients had a mean MMSE score of 21.0 ± 2.1, a global CDR score of 0.8 ± 0.2 and a CDR SB score of 2.8 ± 1.1 at baseline, having significantly greater cognitive impairment compared with the amyloid-positive MCI and CN subjects. At follow-up of 3.3 ± 0.3 years, the mean MMSE score significantly decreased to 16.1 ± 3.2 (*n* = 11, *p* < 0.05), and global CDR and CDR SB deteriorated to 1.3 ± 0.4 (*n* = 11, *p* < 0.05) and 5.5 ± 2.5 (*n* = 11, *p* < 0.05), respectively. In contrast, the four amyloid-positive and nine negative CN subjects had no cognitive impairment on MMSE, global CDR or WMS-R Immediate and Delayed Recall scores at baseline and follow-up. None of the CN subjects progressed to MCI or AD during the follow-up period.

The seven typical positive MCI patients had a mean MMSE score of 26.1 ± 1.4 and a CDR SB of 0.7 ± 0.2 at baseline, similarly to the focal positive and amyloid-negative MCI patients. A mean WMS-R Delayed Recall score in typical positive MCI patients was 1.4 ± 2.6, which was smaller than that in the amyloid-negative MCI patients. Five (71%) of the seven typical positive MCI patients progressed to AD during the follow-up of 3.0 ± 0.2 years (range: 2.5–3.5 years). At follow-up, MMSE scores in typical positive MCI patients decreased to 22.0 ± 0.8 (*n* = 7, *p* < 0.05) and CDR SB scores increased to 2.1 ± 0.9 (*n* = 7, *p* < 0.05). In contrast, none of the four focal positive MCI patients progressed to AD during a follow-up of 3.0 ± 0.1 years. The MMSE and CDR SB scores at follow-up did not significantly differ from those at baseline. None of the six amyloid-negative MCI patients progressed to any dementia. There were no significant differences in MMSE and CDR SB scores between at baseline and follow-up.

### Aβ Deposition

**Figure 1:**
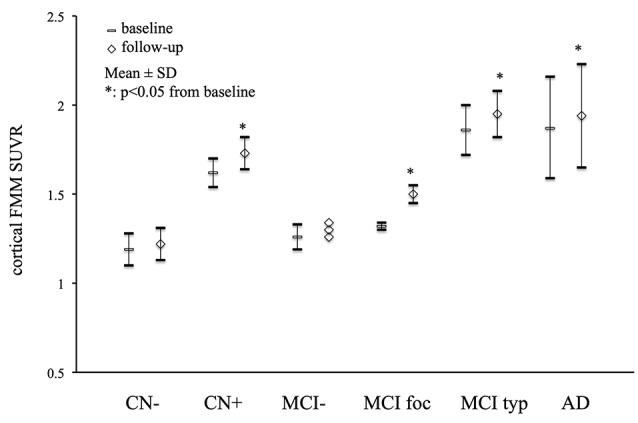
Mean cortical [18F]-flutemetamol (FMM) standardized uptake value ratio (SUVR) at baseline and follow-up in Alzheimer’s disease (AD) patients, typical positive mild cognitive impairment (MCI typ) and focal positive MCI patients (MCI foc), amyloid-negative MCI patients (MCI−), amyloid-positive cognitively normal (CN) subjects (CN+), and amyloid-negative CN subjects (CN−). Data are presented as mean ± standard deviations (SD). *Statistically significant difference from baseline by paired *t*-test (*p* < 0.05).

Mean cortical FMM SUVR values at baseline and follow-up in AD, MCI, and CN subjects are presented in Figure 1. The cortical FMM SUVR in AD patients was 1.87 ± 0.29 (*n* = 11, *p* < 0.01) at baseline, which was higher than that in amyloid-negative CN (1.19 ± 0.09, *n* = 9) or amyloid-negative MCI patients (1.25 ± 0.06, *n* = 6). The typical positive MCI patients had high cortical FMM SUVR of 1.86 ± 0.14 (*n* = 7), being the same high level as AD. In amyloid-positive CN subjects, cortical FMM SUVR was also high (1.62 ± 0.08, *n* = 4), which was slightly lower than AD patient. At follow-up, AD and amyloid-positive MCI and CN subjects had significantly higher cortical FMM SUVR than that at baseline. The cortical FMM SUVR in AD patients increased to 1.94 ± 0.29, which was not different from that in typical positive MCI (1.95 ± 0.13) or CN subjects (1.73 ± 0.09). In focal positive MCI patients, the cortical FMM SUVR increased significantly from 1.32 ± 0.02 at baseline to 1.50 ± 0.05 (*n* = 4, *P* < 0.05). The amyloid-negative MCI and CN subjects had no significant change in cortical FMM SUVR at follow-up.

**Figure 2:**
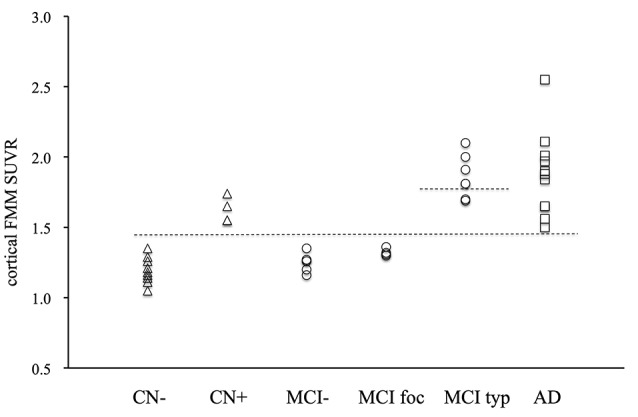
Individual cortical FMM SUVR values at baseline in AD, typical positive MCI (MCI typ) and focal positive MCI (MCI foc), amyloid-negative MCI (MCI−), amyloid-positive CN (CN+) and negative CN subjects (CN−). The upper dotted line indicates the FMM SUVR of 1.81, while the lower line indicates the FMM SUVR of 1.50.

The individual cortical FMM SUVR values at baseline in the AD patients, the typical and focal positive MCI patients, the amyloid-negative MCI patients and the amyloid-positive and negative CN subjects are shown in Figure 2. All 22 subjects with typical amyloid-positive scans had high cortical FMM SUVR of above 1.50. Five of seven typical positive MCI patients had a higher FMM SUVR of above 1.81, all of whom progressed to AD. Although four focal positive MCI patients had a cortical FMM SUVR of below 1.36, all of them had high regional FMM SUVR of above 1.50 in precuneus; two of them had high regional FMM SUVR in another cortical regions, and one had a regional FMM SUVR of 1.40 in the parietal cortical region while the other had 1.37 in the frontal cortical region. Four amyloid-positive CN subjects had a high cortical FMM SUVR of 1.55–1.74.

### Changes in Aβ Deposition

**Figure 3:**
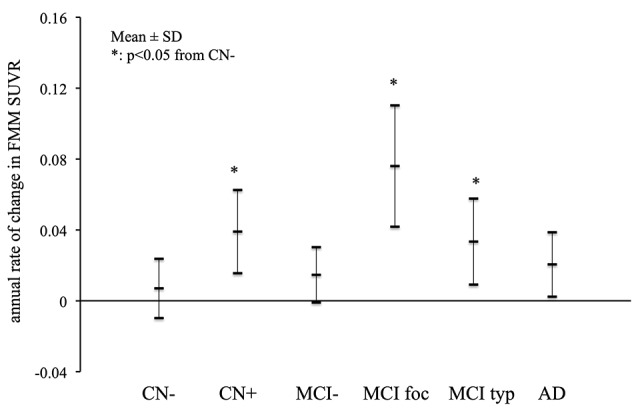
The mean annual rate of change in the cortical FMM SUVR in AD, typical positive MCI (MCI typ) and focal positive MCI (MCI foc), amyloid-negative MCI (MCI−), amyloid-positive CN (CN+) and negative CN subjects (CN−). *Statistically significant difference from CN− by Student’s *t*-test (*p* < 0.05).

The mean annual rates of change in the cortical FMM SUVR in the AD, MCI and CN subjects are shown in Figure 3. The annual rates of increase in cortical FMM SUVR in typical positive MCI patients (0.033 ± 0.024, *n* = 7, *p* < 0.05) and amyloid-positive CN subjects (0.039 ± 0.023, *n* = 4, *p* < 0.05) were significantly greater than those in amyloid-negative CN subjects (0.007 ± 0.016, *n* = 9). The annual increase rate in AD patients (0.020 ± 0.018, *n* = 11) was small, being not significantly different from the amyloid-negative CN subjects. In the focal positive MCI patients in particular, the annual rate of increase was the greatest among these groups (0.076 ± 0.034, *n* = 4, *p* < 0.01). The increase of cortical FMM SUVR was 5.8% per year.

The annual rate of change in regional FMM SUVR for four different cortical regions in AD, typical and focal positive MCI, amyloid-negative MCI and amyloid-positive and negative CN subjects are shown in Table 2. Among these cortical regions, the annual rate of increase in regional FMM SUVR was the greatest in precuneus in focal positive MCI patients (0.097 ± 0.029, *n* = 4, *p* < 0.05), being significantly different from that in the amyloid-negative CN subjects. The increase rate of regional FMM SUVR in the frontal cortical region was also greater in focal positive MCI patients (0.093 ± 0.053, *n* = 4), but not significantly. There was no significant difference in annual rate of change in regional FMM SUVR between these cortical regions in the AD, typical and focal positive MCI, and amyloid-positive CN group.

**Table 2:** Annual rate of change in regional [18F]-flutemetamol (FMM) standardized uptake value ratio (SUVR) in four different cortical regions of AD, MCI and CN subjects.

	LTC	FC	Pre	PC
AD	0.022 ± 0.031	0.034 ± 0.046	0.015 ± 0.038	0.031 ± 0.059
MCI typ	0.036 ± 0.025	0.048 ± 0.034	0.037 ± 0.046	0.049 ± 0.020
MCI foc	0.077 ± 0.031	0.093 ± 0.053	0.097 ± 0.029*	0.053 ± 0.025
MCI−	0.009 ± 0.030	0.033 ± 0.030	0.013 ± 0.051	0.037 ± 0.029
CN+	0.054 ± 0.036	0.041 ± 0.032	0.048 ± 0.038	0.046 ± 0.054
CN−	−0.013 ± 0.028	0.022 ± 0.030	−0.001 ± 0.041	−0.019 ± 0.031

typ, typical amyloid-positive; foc, focal amyloid-positive; +, amyloid-positive; −, amyloid-negative; LTC, lateral temporal cortex; FC, frontal cortex; Pre, precuneus; PC, parietal cortex; Data are presented as means ± SD. *Statistically significant difference from CN- by Bonferroni test (*p* < 0.05).

When the individual annual rate of change in cortical FMM SUVR was correlated to the baseline FMM SUVR in all subjects, there was no significant correlation between them with simple model (*R*^2^ = 0.002, *n* = 41, *p* = 0.73) and inversed U-shape model (*R*^2^ = 0.07, *n* = 41, *p* = 0.23). In contrast, among amyloid-positive patients, the individual annual rate of increase in cortical FMM SUVR had significantly inverse correlation with the baseline FMM SUVR (*r* = −0.44, *n* = 26, *p* < 0.05).

### Comparison Between FMM SUVR and PIB SUVR

**Figure 4:**
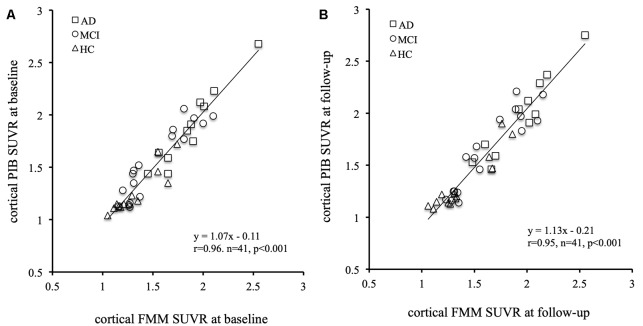
Relationship of cortical FMM SUVR and cortical Pittsburgh Compound-B (PIB) SUVR in individual AD (squares), MCI (circles), and CN subjects (triangles) at baseline **(A)** and follow-up **(B)**. The FMM SUVR is significantly correlated to PIB SUVR at baseline (*r* = 0.96, *n* = 41, *p* < 0.001) and follow-up (*r* = 0.95, *n* = 41, *p* < 0.001).

**Figure 5:**
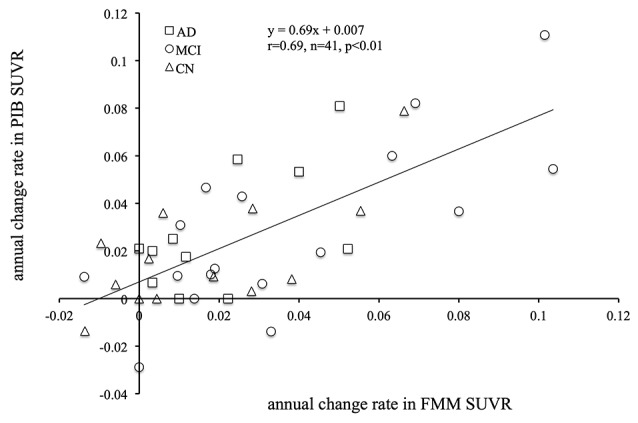
Relationship between annual rate of changes in cortical FMM SUVR and PIB SUVR in AD (squares), MCI (circles), and CN subjects (triangles). Annual rate of change in FMM SUVR is significantly correlated to that in PIB SUVR (*r* = 0.69, *n* = 41, *p* < 0.01).

The mean cortical FMM and PIB SUVRs at baseline, and the annual rate of change in cortical FMM and PIB SUVRs in the same AD, MCI, and CN subjects are shown in Table 3. The baseline value and the annual increase rate of cortical FMM SUVR in each group were not different from those of cortical PIB SUVR. The individual cortical FMM SUVR was significantly correlated with cortical PIB SUVR at baseline (*r* = 0.96, *n* = 41, *p* < 0.001) and at follow-up (*r* = 0.95, *n* = 41, *p* < 0.001; Figure 4). Furthermore, an individual annual rate in change of cortical FMM SUVR was significantly correlated with that in cortical PIB SUVR (*r* = 0.69, *n* = 41, *p* < 0.01; Figure 5).

**Table 3:** Mean cortical FMM and Pittsburgh Compound-B (PIB) SUVRs at baseline and annual rate of change in cortical FMM and PIB SUVRs in AD, MCI and CN subjects.

		[18F]-FMM SUVR		[11C]-PIB SUVR	
	*n*	Baseline	Δchange/year	Baseline	Δchange/year
AD	11	1.87 ± 0.28*	0.020 ± 0.018	1.88 ± 0.36*	0.027 ± 0.024
MCI typ	7	1.86 ± 0.14*	0.033 ± 0.024*	1.91 ± 0.10*	0.033 ± 0.035*
MCI foc	4	1.32 ± 0.02*	0.076 ± 0.034*	1.45 ± 0.06*	0.053 ± 0.036*
MCI−	6	1.25 ± 0.06	0.014 ± 0.030	1.18 ± 0.06	0.008 ± 0.013
CN+	4	1.64 ± 0.08*	0.039 ± 0.023*	1.55 ± 0.15*	0.047 ± 0.018*
CN−	9	1.19 ± 0.09	0.007 ± 0.016	1.14 ± 0.05	0.005 ± 0.010

Δchange/year, annual rate of change; typ, typical amyloid-positive; foc, focal amyloid-positive; +, amyloid-positive; −, amyloid-negative; n, number of subjects; Data are presented as means ± SD. *Statistically significant difference from CN- by Student’s *t*-test (*p* < 0.05).

### Aβ Deposition, Cognition, Age, and APOE Genotype

Of the 26 amyloid-positive patients, the mean cortical FMM SUVR at baseline in the 14 APOEε4 carriers (1.76 ± 0.33, *n* = 14, *p* = 0.77) did not differ significantly from that in the 12 non-carriers (1.73 ± 0.22, *n* = 12). The annual rate of increase in FMM SUVR in the APOEε4 carriers (0.043 ± 0.028, *n* = 14) was greater than that in non-carriers (0.026 ± 0.029, *n* = 12), but the difference was not significant. There was no significant relationship between cortical FMM SUVR at baseline and MMSE score in individual amyloid-positive subjects (*r* = −0.29, *n* = 26, *p* = 0.14). Furthermore, the annual rate of changes in cortical FMM SUVR was not significantly correlated to that of the MMSE score in individual amyloid-positive subjects (*r* = 0.30, *n* = 26, *p* = 0.12) or that of the CDR SB score (*r* = −0.27, *n* = 26, *p* = 0.18). The baseline age was not significantly correlated to the cortical FMM SUVR at baseline (*r* = 0.14, *n* = 26, *p* = 0.48) or the annual rate of increase in cortical FMM SUVR (*r* = −0.14, *n* = 26, *p* = 0.49) in individual amyloid-positive subjects.

## Discussion

We demonstrated that the cortical FMM SUVR in AD, amyloid-positive MCI, and CN subjects increased over follow-up. The annual rate of increase in cortical FMM SUVR was significantly greater in amyloid-positive MCI and CN subjects while it was relatively small in AD patients, and it was the greatest in focal positive MCI patients. A previous [11C]-PIB PET study revealed that MCI patients with high PIB retention had faster increase rates of Aβ deposition, similarly to HC subjects with high PIB retention, but AD patients had a slower rate (Jack et al., 2013). Our previous study demonstrated that the patients with MCI due to AD had greater rates of increase in Aβ deposition during the process of progression to AD, followed by smaller rates of increase at the stage of AD dementia (Hatashita and Wakebe, 2017). These findings indicate that the increase of Aβ deposition does not occur at a constant rate across the clinical stages of the AD spectrum. Aβ deposition could increase faster in MCI patients than in AD patients, and if MCI patients have focal amyloid-positive scans, the Aβ deposition would increase further faster.

In the present study, the annual rate of increase in regional FMM SUVR differed between the cortical regions in the amyloid-positive groups. The increase rate of regional FMM SUVR was the greatest in the precuneus in focal positive MCI patients, followed by the frontal cortical region. Previous [11C]-PIB PET studies have reported that the parietal and frontal cortices and the posterior cingulate showed the most prominent increase in [11C]-PIB uptake in MCI patients (Villemagne et al., 2011; Kemppainen et al., 2014). These findings indicate that Aβ deposition increases faster in precuneus/posterior cingulate in amyloid-positive MCI patients. On the other hand, the AD pathophysiological process has been demonstrated to be temporoparietal and/or precuneus hypometabolism. In the patients with MCI due to AD, we have reported that a regional hypometabolism in the temporal, parietal, and/or precuneus cortices detected by [18F]-fluorodeoxyglucose (FDG) PET is associated to the progression to AD (Hatashita and Yamasaki, 2013). Therefore, we suggest that the faster increase of Aβ deposition, particularly in precuneus, could cause primarily downstream neurodegeneration in the predementia stage.

The annual rate of change in Aβ deposition has been described as providing the estimation for the duration of clinical progression of disease according to individual current Aβ deposition. The time span of disease progression has been estimated at 19.2 years for an individual to go from a PIB SUVR threshold of 1.5 in HC to a PIB SUVR of 2.33 in AD, equivalent to a 0.043 SUVR increase per year (Villemagne et al., 2013). The present study has demonstrated that the estimated time for disease progression to MCI was 6.15 years in amyloid-positive CN subjects to move from the mean cortical FMM SUVR of 1.62 in CN subjects to FMM SUVR of 1.86 in MCI patients based on a mean 0.039 FMM SUVR increase per year. In contrast, we have recently reported that 63% of 16 HC subjects with preclinical AD progressed to MCI within 7 years based on each clinical core criteria of the NIA-AA diagnostic guidelines (Hatashita and Wakebe, 2019). The time of clinical progression that is estimated by the amount and increased rate of Aβ deposition might actually be smaller than what occurs. The time of clinical progression in the preclinical stage of the AD spectrum would vary with APOE genotype, age, education, cognitive reserve and combined brain pathologies, in addition to increase of Aβ deposition.

On the other hand, in the typical positive MCI patients who had almost the same high level of FMM SUVR of 1.86 at baseline as AD, the present study found that the estimation of time for progression to AD dementia was 0.30 years, based on the annual rate of increase in FMM SUVR of 0.033. However, none of these MCI patients actually progressed to AD within 2 years. This implies that the typical positive MCI patients do not progress to AD quickly, even if Aβ deposition reaches the same level as AD. Furthermore, the present study showed that the increased rate of FMM SUVR was not correlated to the decline of MMSE or CDR SB scores in amyloid-positive subjects. In the predementia stage of AD, the increase rate of Aβ deposition does not appear to cause progressive cognitive deterioration directly but rather to trigger downstream AD neuropathological change.

The comparison of the [18F]-FMM PET and [11C]-PIB PET imaging has been studied along the continuum from normal cognitive status to the dementia of AD (Mountz et al., 2015; Lowe et al., 2017). We had already reported that the quantitative measurement of [18F]-FMM PET images, in addition to visual assessment, was consistent with that of [11C]-PIB PET (Hatashita et al., 2014). In this longitudinal study of [18F]-FMM PET and [11C]-PIB PET, we have demonstrated that the individual cortical FMM SUVR was significantly correlated with PIB SUVR at follow-up, in addition to at baseline, in the same AD, MCI, and CN subjects. In addition, the annual rate of change in cortical FMM SUVR was significantly related to that in cortical PIB SUVR. These findings imply that [18F]-FMM PET imaging successfully evaluates the longitudinal assessment of Aβ deposition across the AD spectrum, similarly to a standard approach of [11C]-PIB PET. Therefore, [18F]-FMM PET imaging can reliably detect longitudinal Aβ deposition in the brain and provide a potential prognostic timeframe based on the amount and increased rate of Aβ deposition. Furthermore, it is likely to play a critical role in the development of anti-amyloid therapies by establishing critical periods suitable for intervention along the disease pathway.

Certain limitations of our study should be noted. We conducted a successful longitudinal assessment of Aβ deposition across the AD spectrum during a follow-up period. The quantification was performed by regional FMM and PIB SUVRs normalized to a reference region of cerebellar gray matter. The number of participants included in the assessment was also relatively small, especially for the amyloid-positive CN subjects and focal positive MCI patients.

In conclusion, the [18F]-FMM PET imaging can clarify the longitudinal assessment of Aβ deposition and the increase rate of Aβ deposition across the AD spectrum, similarly to [11C]-PIB PET. The increase of Aβ deposition is faster in the predementia stage of AD and slower in the dementia stage. The amount and increased rate of Aβ deposition could not directly affect a potential prognostic timeframe across AD spectrum.

## Data Availability

All datasets generated for this study are included in the manuscript.

## Ethics Statement

The study was approved by the Ethics Committee of the Mirai Iryo Research Center Incorporation (Tokyo, Japan). All subjects or their caregivers provided written informed consent for participation.

## Author Contributions

YK and DW provided substantial contribution to the acquisition of data. SH and AI provided the analysis of data. YK, AI, DW and SH reviewed the article.

## Conflict of Interest Statement

The authors declare that the research was conducted in the absence of any commercial or financial relationships that could be construed as a potential conflict of interest.
